# Supplementary material for: Comparative Analysis of Transcriptomes of Ophiostoma novo-ulmi ssp. americana Colonizing Resistant or Sensitive Genotypes of American Elm
Source: J Fungi (Basel). 2022 Jun 16;8(6):637. doi: 10.3390/jof8060637 (PMC9224576; doi:10.3390/jof8060637)
Supplement: Supplementary file 1 [file jof-08-00637-s001.zip › Supplementary Table S1-Corrected.pdf]

Supplementary Table S1: Nucleotide sequences designed and synthesized for the RNAi cassettes of each target gene and the modified pAN-7 transformation vector. Annealing sites for PCR primers are indicated.

**Sequence name and length (bp)**	**Nucleotide sequence, forward primer annealing site (underline) for target genes and both forward and reverse annealing sites (bold) in the pAN7 transformation vector**
CYP52P6 RNAi Cassette (1,011)	ACTAGTATCTCTCTGGCCGGAAGTCATCGGCATCCTCGCCATAGAGATCCTTGCGGCGATGCATGGCATACACGCAGTAGCC<br>GACACCCTCGCCTTTGTGTACAAGCACGGGAGACTTGCCATCTGGACCGCCGCCAACAGGCAGGACCGTATCACGCATAGCC<br>TCGCGCGAGTTGACCGGGACGGAGGGGTACAGACGCAGGACTTCTTTGATGACACAGTGGAGGTAGTGCATGTTCTTGAGC<br>TGGGTCTTGGTAGGAGGATCGGCAGCGGGACCAACGCCACATACACTCTCGACCTCCTTGCGCAGACGAGCCAGGACGTGT<br>TGGTGGCGTGCAAGAAGACGGAAGGTCCAGCCAAGACAACAGCCGGTGGTATCTCTGCCAGCAAGCAGCACGTTGAGTGC<br>CTGATCGCGCAACACAGTACGGTCTTGTGTCAAACGGGCTAGGATCTTGATAAACACATCGTGATCTTCATCATCGACAGTC<br>GCCTCTGCAATTTTGGCAGAAGCAGCAGCGTCTTTGCCAAAGTTGGGGCCGAGCGACTTGTCAATAGCCGACTCGACAAACG<br>TATGGCATGTCCGGCACGCATCCCGAAATTCCTTGCTACTCAACGTGCTGCTTGCTGGCAGAGATACCACCGGCTGTTGTCTT<br>GGCTGGACCTTCCGTCTTCTTGCACGCCACCAACACGTCCTGGCTCGTCTGCGCAAGGAGGTCGAGAGTGTATGTGGCGTTG<br>GTCCCGCTGCCGATCCTCCTACCAAGACCCAGCTCAAGAACATGCACTACCTCCACTGTGTCATCAAAGAAGTCCTGCGTCTG<br>TACCCCTCCGTCCCGGTCAACTCGCGCGAGGCTATGCGTGATACGGTCCTGCCTGTTGGCGGCGGTCCAGATGGCAAGTCTC<br>CCGTGCTTGTACACAAAGGCGAGGGTGTCGGCTACTGCGTGTATGCCATGCATCGCCGCA<u>AGGATCTCTATGGCGAGGATG</u><br><u>C</u>CGATGACTTCCGGCCAGAGAGATGGTACC
AmtA RNAi cassette (1,022)	ACTAGTCCTTCTCCTGCGCGCCAAAGGCCATGGCCATGCCGATGCCGTGCATGCCCGACGACACCGTGTGCACGACGTGGGA<br>GTCGCGCGGGCCCAGACCACCGGCTCCGTGGCGATCGGCCGAGTCCATGGAGCCGGTCTCGCCGTCGGCCTCGTTGAGCAC<br>CTCGCGCGTCAGCTCGACATAGTCGTAGGCAAACTCGCCAATCTCGCTGTCGTCGATGCCCAGGATCTCGGCCTCTTCGCTGG<br>CGCGCAGATGCAGGCCGGGGATCATGTTCATCACAAACAGGATCAGGGTGGTGCCGACAAACGAGTAGCAGCCGCCGCTG<br>AAGGAATCGGCCAGCTGGATGCCCAGCTGGGCCCAGTGGTGGTTGAGCCAGCCGCCCTGGATGACCTGGACACCGTCGAG<br>GTGGGCAATGTAGTCGGCCGCGAAGAAGGCTGTGCAAATGTTGCCGACGAGGCCGCCAATGGCGTGCACGGCGAAAATGT<br>CGAGGGCGTCGTCGATGCGCAGCAGGAACTTGAGCTTGGTGGCATAGTTGGCAAATGAGGCGCCGAGCACGCCATAGGGC<br>ACGGCGGCCCAAGCGGGCACGTAGCCAGAACCAGGCGTGATAGCGACGAGGCCGACGGTGTCCAGGTCATCCAGGGCGGC<br>TGGCTCAACCACCACTGGGCCCAGCTGGGCATCCAGCTGGCCGATTCCTTCAGCGGCGGCTGCTACTCGTTTGTCGGCACCA<br>CCCTGATCCTGTTTGTGATGAACATGATCCCCGGCCTGCATCTGCGCGCCAGCGAAGAGGCCGAGATCCTGGGCATCGACGA<br>CAGCGAGATTGGCGAGTTTGCCTACGACTATGTCGAGCTGACGCGCGAGGTGCTCAACGAGGCCGACGGCGAGACCGGCT<br>CCATGGACTCGGCCGATCGCCACGGAGCCGGTGGTCTGGGCCCGCGCG<u>ACTCCCACGTCGTGCACA</u>CGGTGTCGTCGGGCA<br>TGCACGGCATCGGCATGGCCATGGCCTTTGGCGCGCAGGAGAAGGGGTACC

Mad1 RNAi cassette<br>(1,087)	ACTAGTGGAACCGAGACCGTAGCAGAGCTGGTAGGCACCGAGGCGGGGACGGTGGGGACCGACGAGACGACGACGGGAA<br>CCGAGACACTGCCGTAAGGGACCGAGGCGTAGCCAGTGGGGACCGACGAGATGGCAGGGACCGACGAGGACTTGGTGGC<br>GGGGCTGGTAGGGGTGGTCTCGACGGTGGTGGCGGTAGAGGTGGAGGAACTGCAGCCGAAAGAAACGGTAGAGACGGA<br>AACGCTGCAGGTAGTGCTCTTGGGGTGCTTGGGCTGCTTAGGGTAGACAATGGTGCACGCCTTGGCACCACCGCACTTGTCG<br>TTCTTAATGACGCTGCCCTTGCTGGAGCAGGAGCTGGTCTGCTTGCAGGTAGAGCCATCGCCCATGTCGTAGTGGAACTCGA<br>GGTCGCAGTCGAACTCGGTGGTGATGTGGAACTCGGTCACGGAGAAGGAGTCGACGCTCTTGGAGTCCTTGCAGGAGAAC<br>GAGGGGGACTTGTCGGCATCGACGTGGGAGACACCGCGGGCGTATTTGCTGCCGTAGCTGCCGCCGTAGCTGCCGCCGTAG<br>CTGCCGCCGCTGCTGCCACCGTAGCTGCCACCGCTGTTGTCATCGCCGTAGCTGCCACCGTAGCTTCCACCGTAGCTGCCGTC<br>TCCGTGACCGAGTTCCACATCACCACCGAGTTCGACTGCGACCTCGAGTTCCACTACGACATGGGCGATGGCTCTACCTGCA<br>AGCAGACCAGCTCCTGCTCCAGCAAGGGCAGCGTCATTAAGAACGACAAGTGCGGTGGTGCCAAGGCGTGCACCATTGTCT<br>ACCCTAAGCAGCCCAAGCACCCCAAGAGCACTACCTGCAGCGTTTCCGTCTCTACCGTTTCTTTCGGCTGCAGTTCCTCCACCT<br>CTACCGCCACCACCGTCGAGACCACCCCTACCAGCCCCGCCACCAAGTCCTCGTCGGTCCCTGCCATCTCGTCGGTCCCCACT<br>GGCTACGCCTCGGTCCCTTACGGCAGTGTCTCGGTTCCCGTCGTCGTCTCGTCGGTCCCCACCGTCCCCGCCTCGGTGCCTAC<br>CAGCTCTGCTACGGTCTCGGTTCCGGTACC
Hex1 RNAi cassette<br>(1,010)	ACTAGTACGGGATAGTGACAGTGTTGGCGGGAGTCTCAGACTCGCGGCGAACGCCGCGCGAAGGGCTGCTGGCGCGGACA<br>GGCTCGCGGTACTCAGCGACCTCGTTGGAGGGCTCGCGGGCATCCTCGTCGTAGTAACCCATCTTGTTGGATTTGCGGGAAA<br>TGGTTGAAGGGGCGATGATTTCTTGGGTGACCTTAGTCTTGCTGAAAGTGCTGCCCTGGGGACGGACAACAGGAATGTCGA<br>TGGTGGTGTCCTGGTACGATGACTGTTCGGACTGCTCAGCGAAGCGGCCGGTATCAACAGTGGATTCGAAAGAACTAGCTT<br>CCTCACGGTAGCTGGGCTTGAAACGTTTGCGGTATTCGCGCTCGGCAACGTCAATTGCAGAGTCGTGGCTGGAGCGACGGG<br>ACTCAAAGTCTACAGAGGCCTCAAAGGTCGTGTGAGGACGGTTTTCACGAATAAATTCTTGGCGGACACCAGGACGGTAGC<br>GTTCCTCTTCACGAGTGATGCGAACCTCTTCTTCCCGGTAAGGGTAGCTGATGATGTTGGACTCGTTGGCAACGTGAGAAGA<br>GTATTGACCTTCCCGTCCGGGCCGTTCGTGGTGATGAGGTAGCTGTCCAGCCACGACTCTGCAATTGACGTTGCCGAGCGCG<br>AATACCGCAAACGTTTCAAGCCCAGCTACCGTGAGGAAGCTAGTTCTTTCGAATCCACTGTTGATACCGGCCGCTTCGCTGAG<br>CAGTCCGAACAGTCATCGTACCAGGACACCACCATCGACATTCCTGTTGTCCGTCCCCAGGGCAGCACTTTCAGCAAGACTAA<br>GGTCACCCAAGAAATCATCGCCCCTTCAACCATTTCCCGCAAATCCAACAAGATGGGTTACTACGACGAGGATGCCCGCGAG<br>CCCTCCAACGAGGTCGCTGAGTACCGCGAGCCTGTCCGCGCCAGCAGCCCTTCGCGCGGCGTTCGCCGCGAGTCTGAGACTC<br>CCGCCAACACTGTCACTATCCCGTGGTACC
CYP570AA4 RNAi<br>cassette<br>(1,041)	ACTAGTCGCACGTTCATGTCGCTCTGAATAAAGAATGCAGCGTTGAAGGATTTCCACGGGTGGGCAGGGTCGACGAGAACA<br>ATATCAAAGCGGCGCAGCAGCTCGACATAGGTCTTGTTAAGTTCAATCATGGCAATATTCTTTCCAAGACACTCGTAGCGGCT<br>ACCACCACCAAAGTCAAGCATGACCGTGTTGTCCATCAGCTTGTACCGCTCAGTGGGAATGTCCATCCAGCGATCTGGGCGG<br>AACAGGTTGGCATCTTCACCAAAGATAGTCTTGTTGCGCATAACGGGCCATGGTGCCCAGGCGACAGACGTACCAGCAGGC<br>ACGCGGTAGCCACAAATTTCGTCATCAGTCTTTGAGACCTTAGGAAGAAGACCGGTGGCAGGTGGCCACATGCGGAGACCT<br>TCGCGGATAACAGCTTGAAGAAAGGGGAACTGGCGAGCTTCGGCATCGGTAATCGGCGAGCTGATGCGGCCAGCACGGAT

	ACCCTCATCGATTTCCTTTTGCAGGTGCACATAAACGTGTGGGTTTGTAATGAGGTAGAGCAAGGTTGAACGTATGGCCGTG<br>GCTGATGTGTCGGAGCCAGCAATAATCTGGACGACGGCCCTTTCTTCAAGCTGTTATCCGCGAAGGTCTCCGCATGTGGCCA<br>CCTGCCACCGGTCTTCTTCCTAAGGTCTCAAAGACTGATGACGAAATTTGTGGCTACCGCGTGCCTGCTGGTACGTCTGTCGC<br>CTGGGCACCATGGCCCGTTATGCGCAACAAGACTATCTTTGGTGAAGATGCCAACCTGTTCCGCCCAGATCGCTGGATGGAC<br>ATTCCCACTGAGCGGTACAAGCTGATGGACAACACGGTCATGCTTGACTTTGGTGGTGGTAGCCGCTACGAGTGTCTTGGAA<br>AGAATATTGCCATGATTGAACTTAACAAGACCTATGTCGAGCTGCTGCGCCGCTTTGATATTGTTCTCGTCGACCCTGCCCAC<br>CCGTGGAAATCCTTCAACGCTGCATTCTTTATTCAGAGCGACATGAACGTGCGGGTACC
Cox2 RNAi cassette<br>(1,021)	ACTAGTCAAAGACTTTGCGAAGTGCCTTGTAGCTTTTATCAATTTGCATCTTTGGGATAAAGCTCATCCATGAAGGAAGGACG<br>CTGGACATTTGCATCGTTATGTTGATGGCGTCGTTGAACTTGAGGAATATGTCTACAAGCTCGGGATGGTCGTATGCATCGG<br>CACCGAGCATGCAACGCGCGCTGACAGTCGTGCTGAATTTGTCCATATAGCCGCTCAATGTCTCGTGGGTGGCAGAAGTCAT<br>GCCTGTCGGAACCAGTGGGTTGTTGGCAAGAAAGTGCTTGGCGGCGGCTTCCATAGTGCTGCCGAAAGCGCGAATGTTGGC<br>TGGGTCGCCGAGGTGTGTCCGGAGTAGAGTTGCGTGAAGGTTGGTGGAAAATGTTTCCTTTCCGAGGAAGATGGGTGCTCC<br>GAGAATCTCAAAGAACTGGTCAATGTTAAAGTAGACTTGGTATGGGTTGTCATCCTGCATCATCTGGAGATCGTGGGGGTG<br>GGACAGAACGTAATGGGTCTGTGGTCCGAGATGAATGCCAAAAGCCCTGGATGGACTGTTGCGGTTTCCCAGCAGCTGAAA<br>TCCTTTGACAAGAACAGACTTGGCGTCTTGCTGAAAGGCCCGGAGCACCCATCTTCCTCGGAAAGGAAACATTTTCCACCAAC<br>CTTCACGCAACTCTACTCCGGACACACCTCGGCGACCCAGCCAACATTCGCGCTTTCGGCAGCACTATGGAAGCCGCCGCCA<br>AGCACTTTCTTGCCAACAACCCACTGGTTCCGACAGGCATGACTTCTGCCACCCACGAGACATTGAGCGGCTATATGGACAA<br>ATTCAGCACGACTGTCAGCGCGCGTTGCATGCTCGGTGCCGATGCATACGACCATCCCGAGCTTGTAGACATATTCCTCAAGT<br>TCAACGACGCCATCAACATAACGATGCAAATGTCCAGCGTCCTTCCTTCATGGATGAGCTTTATCCCAAAGATGCAAATTGAT<br>AAAAGCTACAAGGCACTTCGCAAAGTCTTTGGGTACC
pAN7-1 transformation<br>vector<br>(3,024)	GGATCCGGGGTTTAACGGTCCCCATGTCAACAAGAATAAAACGCGTTTTCGGGTTTACCTCTTCCAGATACAGCTCATCTGCA<br>ATGCATTAATGCATTGACTGCAACCTAGTAACGCCTTCAGGCTCCGGCGAAGAGAAGAATAGCTTAGCAGAGCTATTTTCAT<br>TTTCGGGAGACGAGATCAAGCAGATCAACGGTCGTCAAGAGACCTACGAGACTGAGGAATCCGCTCTTGGCTCCACGCGAC<br>TATATATTTGTCTCTAATTGTACTTTGACATGCTCCTCTTCTTTACTCTGATAGCTTGACTATGAAAATTCCGTCACCAGCCCTG<br>GGTTCGCAAAGATAATTGCATGTTTCTTCCTTGAACTCTCAAGCCTACAGATCTGGGGTAAGAAGCGTGAAAGAGAAGAGCA<br>GAAGCGTAGAAGGGTAGAAGAGTGAAAGAGAAGAAGAACAAAAAAGAAGAAGAGTAGAAGAGTAAAAAAAGAAGAATA<br>GAAGACAGACACTTGTTATCGAGGCTATCTGATAACTTTGATCATGAGCTTTTCGATGAGATCTAGTCCACAACGGCAGCAA<br>CACCTACTCAGTAGCATAATTTATGATAATTTGGCGAGCAATAGAGCCTGTACAGTCAGTTGTCTCGCGAGCCAAGGTTGCTA<br>TCTTTCCACTTTATGCGTAATTTATTTTCTCAAAATGATCCTCAGTCTTTCCCTTTATTCTTCTGGGCACGGCGAAACCGACAGT<br>CGAAAGAACTGGCATACAGTAAGAATTAATTATGCAAACGACAAGCAGTGCAAATAGCAGAAACACCAAGGTCGGGGAATT<br>GGAGCATGGCATCTCAGTATCAGGTCATTTCCAATGCAACGTGCTCTGGCCGGCCTCTATTAGAAAAGTGCTGCCACTCAAG<br>CCACTCACAGGGAAGGGCCGCCGACGCAGGTGAGATATGAGGGGAATAATTGATGCAGCGCAGGCTAGTATTATCTTATTA<br>TTCTATATTGTGCTCCGCTATGCCTGCATTATTTGCTCCTTCCTTCTTGTATTGCTGCTATTGACAAAGCCCAAGCAAGACACGT

CGCCATGCAGACAAGCCTCTACTCTTTTCCAGTTGCTCTCTTTAGTTCTGCATCTGCTGTATATTGCTTATATTCTTGATACGAA
TACCTATATTGTACTCGATTCTATTCCCGGCGAGCCCGATGTCGCCGAACGCCTTATGACGTATTTACATTGCAGCTCGTTAAT
TCCTCATCTCTAATATCTAATATCTAGTATCTAATATCTTATCTACATCCACCCAGTATCTCATCATCCGGTCTCTTTGCGGTGTT
TCTGTCATACTATCACACGGGGCTGTAAAGTGCTACGAGATACTGCGGGATGGTGATCATGCAATTCGGAAAGCGCGCTTTG
AAAGTCGTAAAGTCGCAAAAGTCAATCAGACTAAAGAGGCAACTACGACGCGCGCGCTCCTTCTCAAATTCCAAGAATCAGC
AGACACCTACACCCACGCAACTGTCGGCCTGCCTTTTAGCGAACAGCGAGCAACAGCTGTCACTCCTCAGCATCCCGCCAAAC
AAAAAAGTCTCGTGTTCCCTACAGCTGTCTCGACTCTGCCAGCTCCAATTATACCCAGTATTCTCTACGTAAATAGTGGCGTG
AATGCCTCCTAAATACACCGTCCCCCAGATCGTCCCTCTCAACCGATCGTTTCTCTTTTGGGCATAGCTCTGTCGGGAAGCTC
ACACGGTAGCTTTATTAAAAATAAATAAATTCTTTTATTCTTCGACTTGTGCGTGGAGTCCAGCTCGACGACATCATACGTTCC
ACAATGCGTCCTTGTCTCTAGTCCTTTTCTCAAGTCGCTGCGAGTCTCTCTACCACTGCTGTACGACTATCTTCTCATACTGCAT
CGCCGTTCGACCGTTTGTGGCAGTAGCTTGCCTCTGCCCGCTATCGTCCCCTCCTTCCCTCTCCTCTCCTCTCCTCTAGCTCTTTC
CTCTTTCCTCTTTCAAGATTGTATCGTCTCCACCTCCGTTCCTCCTTTGCCCTTTGCTCTTGCTCTTACTCTTTTCTCTGTGTGCCT
CGCACTTGCCTACTCCAATTCGCCGTACTCCTCTACTACTACTCCTTTGGGAAAGAGCTGGCAAAAGAGCTGTTGCTAGCCTCT
AACTCACCAACGGTAATACGTCATGAGCTCTTGTAATTACTAGCTTATTCAGAACATATCGTATTTAGTGCAGGGAGAAGTAC
TAACGATGATACTATTATTTCTTGCCCCTCGTAGCTTTTCAATACTATGCTGCTCCAATATCACCGTGGAGACGTCCCTCCCAG
CTCCTCCTCGTGTCTCCCAACTCCCAACAGGAGTTCTTT**CGGGTTCGTCTCCTCTTTCA**GGTCCATCTCTCCTTCCCTTCCGAGT
ATATATAGTCGACCTCGCCACCTCTTGGACAATTCTTTTGTCTCGTCTCATATCTCATCTCATCAACTCATCTTCCCTCTTCTCCT
CTCTTCTCTACAATTACAATCTACATCTAAAGGTTATAGCTTCTTATACCAACTTACTACCATAACCACCTCTACCTTACACATCC
GTCAATACTAGTATCGATGGCGCGCCATGCGAAGCCCGGGGCGGCCGCGGTACCAACGGTCCCCATGTCAACAAGAATAAA
ACGCGTTTTCGGGTTTACCTCTTCCAGATACAGCTCATCTGCAATGCATTAATGCATTGACTGCAACCTAGTAACGCCTTCAGG
CTCCGGCGAAGAGAAGAATAGCTTAGCAGAGCTATTTTCATTTTCGGGAGACGAGATCAAGCAGATCAACGGTCGTCAAGA
GACCTACGAGACTGAGGAATCCGCTCTTGGCTCCACGCGACTATATATTTGTCTCTAATTGTACTTTGACATGCTCCTCTTCTT
TACTCTGATAGCTTGACTATGAAAATTCCGTCACCAGCCCTGGGTTCGCAAAGATAATTGCATGTTTCTTCCTTGAACTCTCA**A**
GCCTACAGAAACCCCAAGCTT
