# Supplementary material for: Comparative Analysis of Transcriptomes of Ophiostoma novo-ulmi ssp. americana Colonizing Resistant or Sensitive Genotypes of American Elm
Source: J Fungi (Basel). 2022 Jun 16;8(6):637. doi: 10.3390/jof8060637 (PMC9224576; doi:10.3390/jof8060637)
Supplement: Supplementary file 1 [file jof-08-00637-s001.zip › Supplementary Table S2-Corrected.pdf]

Supplementary Table S2: Primers used for the qPCR amplification of candidate genes and reference genes to assess expression levels, and PCR primers for the confirmation of a complete RNAi cassette in positive transformants.

Candidate and reference genes	Oligonucleotide sequence (5' – 3')	Primer T_m (°C)	Amplicon size (bp) and T_m (°C)
CYP52P6	qCyP52P6-F1: AGGATCTCTATGGCGAGGATGC	61.5	165, 81.3
	qCyP52P6-R: GGTAACCCCAGCCAATGTTCTTG	61.9	
AmtA	qAmtA-F3: ACTCCCACGTCGTGCACA	61.5	99, 85.5
	qAmtA-R: GCAGCGGGATGTTGTTGCT	61.6	
Mad1	qMad1-F: CTTACGGCAGTGTCTCGGTTC	61.0	167, 87.4
	qMad1-R: GGGAACAGGAACAGAGACCGA	61.4	
Hex1	qHex1-F: AAGATGGGTTACTACGACGAGGAT	60.9	176, 87.8
	qHex1-R: TGCAGGATCAGGATGTCACCA	61.2	
CYP570AA4	qCyP570A4-F: GCGCCGCTTTGATATTGTTCTC	60.9	123, 80.2
	qCyP570A4-R: CTCTCAAAAACGGCCTCAATGC	60.7	
Cox2	qCox2-F2: TCAAGTTCAACGACGCCATCAA	61.1	113, 82.0
	qCox2-R2: CATGGCAGAAAGTCCAGCAGA	60.6	
Actin	Act-143-F: CGGAGAAGGACTCGTACGTTG	64.5	105, 82.44
	Act-248-R: TCCATGTCGTCCCAGTTGGTA	62.6	
β-tubulin	Bt-827-F: GTGGCTCGTACTCTTTCCGTG	64.5	127, 84.0
	Bt-954-R: ACGGAAGATAGCAGAGCAGGT	62.6	
Chromosome protein 1	Psm1-166-F: CACCTCCGTTCCAGTCAACT	62.4	147, 84.37
	Psm1-313-R: CATCACCGGCATCGTCATCT	62.4	
For amplification of RNAi cassette	RNAI PCR-F: CGGGTTCGTCTCCTCTTTCA	62.4	Varies with insert
	RNAi PCR-R: GCTTGGGGTTTCTGTAGGCT	62.4	(1,550 to 1,650)
