# Supplementary material for: Comparative Analysis of Transcriptomes of Ophiostoma novo-ulmi ssp. americana Colonizing Resistant or Sensitive Genotypes of American Elm
Source: J Fungi (Basel). 2022 Jun 16;8(6):637. doi: 10.3390/jof8060637 (PMC9224576; doi:10.3390/jof8060637)
Supplement: Supplementary file 1 [file jof-08-00637-s001.zip › Supplementary Table S3.pdf]

Supplementary Table S3: Primers used to amplify specific gene fragments for producing and verifying OSCAR deletion mutants of *Ophiostoma novo-ulmi* ssp. *novo-ulmi.*

**Primer**	**5'-3' Primer sequence***	
Oao1F_-1031_attB2r	***GGGGACAGCTTTCTTGTACAAAGTGGAATACTGGCGGATTCTTCGACTC***	Primers used for amplification of 1kb of the downstream region of *aox1* with site-specific recombination to produce the deletion construct
Oao1R_-3_attB1r	***GGGGACTGCTTTTTTGTACAAACTTGTCGAATTATTTTTGGTCGCTGAC***	
Oao1F_2507_attB4	***GGGGACAACTTTGTATAGAAAAGTTGTTGTGGTCAGTAGTGGACAACTG***	Primer used for amplification of the upstream region of *aox1* with site-specific recombination to produce the deletion construct
Oao1R_3632_attB3	***GGGGACAACTTTGTATAATAAAGTTGTCTTGTCATTTGCCATACGCGG***	
Oao1F_236<br>Oao1R_840	ACCTCGACCACAACCTCAAAG<br>ATGAGACAACCGGTGGAAGAG	Primers used for amplification of *Δaox1* fragment
Bct2F_-1050_attB2r	***GGGGACAGCTTTCTTGTACAAAGTGGAA***TTCCCAAACGGGCTCTATTC	Primers used for amplification 1kb of the downstream region of *bct2* with site-specific recombination to produce the deletion construct
Bct2R_-1_attB1r	***GGGGACTGCTTTTTTGTACAAACTTGT***AATGAGGGAAAATGGCTATG	
Bct2F_1890_ attB4	***GGGGACAACTTTGTATAGAAAAGTTGTT***TTTTGTTATTCTCTCATTGCC	Primer used for amplification of the upstream region of *bct2* with site-specific recombination to produce the deletion construct
Bct2R_2903_attB3	***GGGGACAACTTTGTATAATAAAGTTGT***GGGTCTGTCGCACATTATC	
Bct2F_539<br>Bct2R_1111	TCCACCTCCACATTCAAGACG<br>AAACCCATCTTTTGCTGACGC	Primers used for amplification of *Δbct2* fragment
Opf2F_-953_attB2r	***GGGGACAGCTTTCTTGTACAAAGTGGAA***AGGCAGAATCGGACCGAGACGG	Primers used for amplification 1kb of the downstream region of *opf2* with site-specific

Opf2R_-1_attB1r	***GGGGACTGCTTTTTTGTACAAACTTGT***GGTGGGAGTTCAGAGGTTGAAG	recombination to produce the deletion construct
Opf2F_2446_attB4	***GGGGACAACTTTGTATAGAAAAGTTGTT***TTGCTTATGCCAGGGACGGACC	Primer used for amplification of the upstream region of *opf2* with site-specific recombination to produce the deletion construct
Opf2R_3468_attB3	***GGGGACAACTTTGTATAATAAAGTTGT***ACCATTTTGCCTCCATGGAAGC	
Opf2R_254<br>Opf2R_873	TTGCTCCAACTTCGGGTCTAC<br>GTTCGCGTATGGGTCATTTGG	Primers used for amplification of *Δopf2* fragment
